# Genome analysis and genetic transformation of a water surface-floating microalga *Chlorococcum* sp. FFG039

**DOI:** 10.1038/s41598-019-47612-8

**Published:** 2019-08-01

**Authors:** Yoshiaki Maeda, Daisuke Nojima, Miki Sakurai, Tatsuhiro Nomaguchi, Momoko Ichikawa, Yuki Ishizuka, Tsuyoshi Tanaka

**Affiliations:** 1 Division of Biotechnology and Life Science, Institute of Engineering, Tokyo University of Agriculture and Technology, 2-24-16 Naka-cho, Koganei, Tokyo 184-8588 Japan; 2 Department of Advanced Science and Engineering, Graduate School of Advanced Science and Engineering, Waseda University, 3-4-1 Okubo, Shinjuku-ku, Tokyo 169-8555 Japan

**Keywords:** Comparative genomics, Genetic engineering

## Abstract

Microalgal harvesting and dewatering are the main bottlenecks that need to be overcome to tap the potential of microalgae for production of valuable compounds. Water surface-floating microalgae form robust biofilms, float on the water surface along with gas bubbles entrapped under the biofilms, and have great potential to overcome these bottlenecks. However, little is known about the molecular mechanisms involved in the water surface-floating phenotype. In the present study, we analysed the genome sequence of a water surface-floating microalga *Chlorococcum* sp. FFG039, with a next generation sequencing technique to elucidate the underlying mechanisms. Comparative genomics study with *Chlorococcum* sp. FFG039 and other non-floating green microalgae revealed some of the unique gene families belonging to this floating microalga, which may be involved in biofilm formation. Furthermore, genetic transformation of this microalga was achieved with an electroporation method. The genome information and transformation techniques presented in this study will be useful to obtain molecular insights into the water surface-floating phenotype of *Chlorococcum* sp. FFG039.

## Introduction

Microalgae have been recognized as promising hosts for production of various types of valuable compounds^1,2^. For instance, it has been reported that microalgae produce neutral lipids useful for biofuel application^3^, fatty acids such as polyunsaturated fatty acids (PUFA) used as nutrient supplements for humans, and also as feeds for aquaculture^4,5^, pigments like β-carotene, astaxanthin and lutein^6^, and proteins such as antitoxins^7^. The advantages of microalgae are its high productivity due to high growth rate, and photosynthetic property that contributes towards decreasing CO_2_ emission. However, there still exist issues that need to be addressed before using microalgae for industrial application. One of the critical issues is harvesting and dewatering, which are known to be very costly and are energy-consuming steps in the entire process^8,9^. Therefore, instead of conventional centrifugation and filtration, a number of alternative harvesting methods, mainly based on the flocculation phenomenon have been proposed^10^.

In contrast, we have proposed a unique harvesting system using microalgal strains that spontaneously float on the water surface^11,12^. These microalgae (tentatively identified as *Botryosphaerella* sp. and *Chlorococcum* sp.) form robust biofilms floating with gas bubbles. The floating biomass was readily harvested from the water surface, and showed less moisture content than that harvested by centrifugation^11^. This suggested that these water surface-floating microalgae have potential to overcome the bottlenecks of micoalgal production described above. However, to our knowledge, there are very few studies on water surface-floating microalgae^13^. Molecular mechanisms underlying the water surface-floating phenotype remain elusive, and no studies have associated any genes with this phenotype as yet.

Over the past decade, various next-generation sequencing techniques have emerged. These techniques allow us to analyse whole genome sequences of non-model organisms easily and cost-effectively. The obtained genome information could provide attractive targets for reverse genetics studies in the future, in which their expression levels are modulated via genetic engineering, and the resulting phenotypic consequences are observed^14^. We expect that this approach is one of the ways to elucidate the water surface-floating mechanisms of microalgae. To achieve this goal, it is essential to establish a genetic transformation technique for these organisms. Genome information is useful not only for screening the target genes that are potentially involved in biofilm formation and floating behaviour, but also for improvement of the established transformation technique by providing the sequences of expression elements such as endogenous promoters.

In this study, we first established the genetic transformation technique based on electroporation for a water surface-floating microalga, *Chlorococcum* sp. FFG039^12^. A neomycin-resistant gene was expressed with heterogeneous promoters in the microalga. Gene retention in the resulting transformant clones were examined by polymerase chain reaction (PCR) and Southern hybridization. Next, the *Chlorococcum* sp. FFG039 genome was analysed with SMRT sequencing from Pacific Biosciences (PacBio). Functional annotation, and comparative genomics with other green microalgae that do not show water surface-floating phenotype, were carried out. From the outcomes of the comparative genomics, we found several gene families that are unique in the *Chlorococcum* sp. FFG039 genome, and potentially involved in biofilm formation. These genes will be attractive targets in future for reverse genomics study aiming to elucidate water surface-floating mechanisms.

## Results and Discussion

### Genetic transformation of *Chlorococcum* sp. FFG039

We consider that both the genome analysis and establishment of transformation technique are useful not only for understanding the molecular mechanism of water-surface floating phenotype but also for future genetic engineering towards improved production of valuable compounds in the microalga. First, we attempted to establish the genetic manipulation technique for *Chlorococcum* sp. FFG039. We tested its sensitivity to an G418, which is an aminoglycoside antibiotic agent as with neomycin, for screening of positive clones. No colony was formed on the agar medium containing 20 µg/ml G418 (Supplementary Table S1). In liquid medium, addition of 100 µg/ml G418 resulted in almost complete inhibition of cell growth (Supplementary Fig. S1). We employed these concentrations in our subsequent experiments.

Next, we studied the electroporation conditions with the plasmids expressing *neomycin phosphotransferase II* (*nptII*), which contained a promoter of the 35 S ribosome subunit derived from cauliflower mosaic virus (CaMV) (pSP-NPT/CaMV) or that of RNA polymerase *α* subunit derived from a diatom, *Fistulifera solaris* (pSP-NPT/rpoA)^15^. The antibiotic G418 and the selection marker *nptII*, which encodes the enzyme to phosphorylate and inactivate aminoglycoside antibiotics like G418, have long been employed for transformation of plants^16^ and microalgae^17,18^. Promoter of the CaMV 35 S ribosome subunit was previously used for expression of a drug-resistant gene in the green microalga *Chlamydomonas reinhardtii*^19,20^, and thus we first selected this promoter for expressing *nptII* in the green microalga *Chlorococcum* sp. FFG039. In addition, to examine whether it is possible for heterologous promoters derived from phylogenetically distant microorganisms to work in *Chlorococcum* sp. FFG039, we employed the promoter of the diatom, which we have long studied for biofuel production^15^. Endogenous promoters were not used as the genome sequence had not been analysed, when we established the transformation technique. We tested various experimental conditions by changing the applied voltage (1.0~10.0 kV/cm), amount of the plasmid (1.0~10.0 μg to 2.5 × 10^7^ cells), and gap between the electrode in the cuvettes (Supplementary Tables S2–S4, respectively), and counted the number of the G418-registant colonies which were formed under each conditions. The optimized conditions were determined as follows, applied voltage: 2.0 kV/cm, DNA quantity: 2.5 µg, gap between the electrodes: 0.4 cm. The transformation frequencies for both plasmids were approximately 5 × 10^−5^. Retention of the target gene *nptII* in the transformants cells were confirmed by PCR using *nptII*-specific primers. No band was observed from wild type (Supplementary Fig. S2(a)), while specific bands were confirmed from both transformants (Supplementary Fig. S2(b,c)). Then, southern hybridization was performed to confirm the insertion of *nptII* in the nuclear genome of the clone harbouring pSP-NPT/CaMV (Supplementary Fig. S3). Besides the 2 plasmids mentioned above, we confirmed that transformation of *Chlorococcum* sp. FFG039 was possible with the plasmid containing the promoter of histone 4 derived from the diatom *F. solaris*^15^ (Supplementary Fig. S3). The transformant clones maintained the water surface-floating phenotype. We cultivated a total of 61 transformant clones (57 clones with pSP-NPT/CaMV and 4 clones with pSP-NPT/rpoA), from which *nptII* was detected by PCR, in the culture medium containing 100 µg/ml G418. After 2 weeks, 58 clones among 61 showed better growth than wild type. We further maintained the clones for approximately 5 months, and found that 6 clones (5 clones with pSP-NPT/CaMV and 1 clones with pSP-NPT/rpoA) retained the G418-resistant phenotype. We then confirmed the retention of the *nptII* gene from one clone of each transformant by performing PCR (Supplementary Fig. S2). These results indicate stable expression of a heterogeneous gene in *Chlorococcum* sp. FFG039, for at least 5 months using heterogeneous promoters.

### Assembly and functional annotation of *Chlorococcum* sp. FFG039 genome

**Figure 1:**
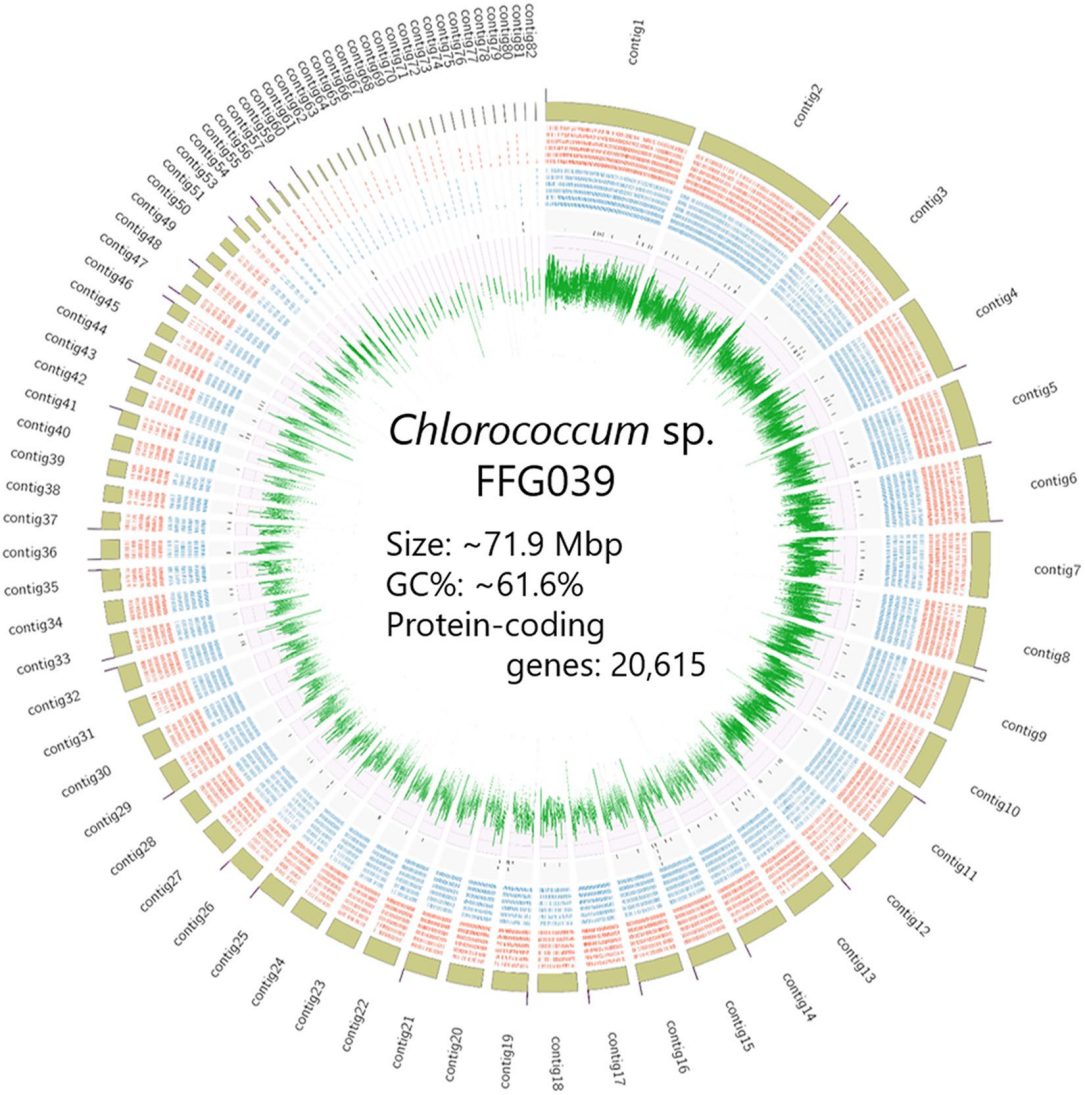
Circular view of the nuclear genomic landscape of *Chlorococcum* sp. FFG039. Eighty contigs and the telomeric repeat sequences (CCCTAAA) detected at the end of the contigs are represented with yellow green tiles and the pink bars located on the contig tiles, respectively. Genes on positive and negative strands are represented with red and blue tiles, respectively. Genes in the unique family identified with comparative genomic study are represented with black tiles. Green lines represents GC content every 3 kb. Contigs 52 and 58 correspond to chloroplast and mitochondrial genomes, and thus are not shown in this figure.

Genome information is useful for obtaining molecular insights into the water surface-floating phenotype of *Chlorococcum* sp. FFG039. In addition, it is expected that the transformation technique for this microalga could be improved if the sequences of expression elements such as endogenous promoters were revealed. We analysed the genome of *Chlorococcum* sp. FFG039 with SMRT Sequencing from PacBio, which generated ~12.3 Gb in ~1.7 × 10^6^ reads. Assembly of these reads generated 82 contigs ranging from ~19.7 kb to ~5.5 Mb with N50 of ~1.8 Mb. The number of the reads that overlap the contigs were calculated, and depth was determined to be 109. We identified 80 contigs corresponding to the nuclear genome (~71.9 Mb in total, Fig. 1), while 1 contig (~190.4 kb, Supplementary Fig. S4) corresponding to a chloroplast genome and another one corresponding to a mitochondrial genome. In the contig for mitochondrial genome, almost identical sequences were repeated twice, probably due to assembling error. Therefore, we manually excluded the repeating sequence to generate a new contig (~36.7 kb, Supplementary Fig. S5). Among the 80 contigs corresponding nuclear genome, 35 contigs had a telomeric repeat sequence (CCCTAAA or complementary TTTAGGG)^21^ at either end (Fig. 1), whereas none of the contigs had it at both ends, suggesting that the contigs presented in this study could be further assembled into longer scaffolds for future studies.

As a result of genome annotation, 20,615 genes were predicted in the nuclear genome. As compared to the model green alga *C. reinhardtii*, *Chlorococcum* sp. FFG039 showed higher gene density (Table 1, 1.36 times more genes in the genome and 0.6 times in size). The contigs corresponding to the genomes of chloroplast and mitochondrion were analysed with BLASTn. The chloroplast genome of *Chlorotetraedron incus* (GenBank: KT199252.1, query cover: 28%, identity: 89%), and mitochondrial genome of *Neochloris aquatic* (GenBank: KJ806271.1, query cover: 37%, identity: 84%) showed the highest similarities to these contigs. Supplementary Figures S4 and S5 show the landscapes of the organelle genomes of *Chlorococcum* sp. FFG039, which were estimated using the chloroplast and mitochondrial genomes of *Chlorotetraedron incus* and *Neochloris aquatic* as reference sequences on GeSeq pipeline.

**Table 1:** General features of nucleic genomes of green algae.

	*Chlorococcum sp*. FFG039	*Chlamydomonas reinhardtii*^22^	*Ostreococcus tauri*^23^	*Chlorella variabilis*^24^	*Volvox cateri*^25^
Genome size (Mb)	72	121	13	46	138
GC content (%)	62	64	58	67	56
Predicted protein-coding genes	20,615	15,143	8,166	9,791	14,520

### Comparative genomics of *Chlorococcum* sp. FFG039 and other green algae

**Figure 2:**
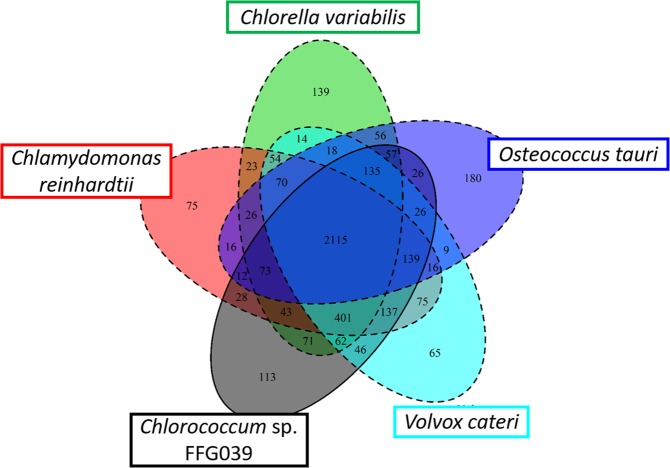
Venn diagrams of shared/unique gene families of *Chlorococcum* sp. FFG039, *Chlamydomonas reinhardtii*, *Osteococcus tauri*, *Chlorella variabilis*, and *Volvox cateri*.

To characterize protein functions encoded in the nuclear genome of *Chlorococcum* sp. FFG039, gene families (retrieved from the domain database Pfam) were searched with InterProScan, and 3,484 gene families (containing 13,328 genes) were detected. These detected gene families were then compared to those in other green algae, namely, *C. reinhardtii*^22^, *Osteococcus tauri*^23^, *Chlorella variabilis*^24^, and *Volvox cateri*^25^. Phylogenetic relationship of these microalgae is shown in Supplementary Fig. S6. From the gene families of *Chlorococcum* sp. FFG039, 113 gene families (containing 168 genes) were identified as unique families (Fig. 2 and Table S5). A survey of the annotated genes revealed several candidate genes which might be related to the floating phenotype, as described below.

GENE_00018951-RA encodes protein containing a unique gene family, jacalin-like lectin domain (PF01419). This domain was found in some bacterial biofilm-associated proteins from *Vibrio cholera*^26^, including biofilm-associated protein 1 (Bap1) and rugosity and biofilm structure modulator C (RbmC). It is likely that these bacterial proteins are secreted from the cells, and facilitate adhesion by binding to carbohydrates in the biofilm. Likewise, the protein encoded by GENE_00018951-RA might be involved in biofilm formation of *Chlorococcum* sp. FFG039 with the aid of the lectin domain. Interestingly, a prediction pipeline of subcellular localizations in eukaryotes, DeepLoc^27^ estimated that this protein is soluble and extracellular. Some other proteins, which may also be involved in cell adhesion and aggregation during biofilm formation were detected as a unique family in *Chlorococcum* sp. FFG039. The protein encoded by GENE_00012902-RA contains cellulose binding domain (PF00942). This protein was also predicted to be soluble and extracellular by DeepLoc pipeline. Some of green algae can possess cell walls containing cellulose like those found in terrestrial plants^28^. Although the cell wall structures of *Chlorococcum* spp. have not been fully elucidated, Miller reported that vegetative cells of *Chlorococcum oleofadens* could have a cell wall containing cellulose^29^. Therefore, the protein encoded by GENE_00012902-RA has a potential to be associated with surface of cell walls. In addition, five proteins were found to contain sushi repeat (SCR repeat) domain (PF00084), which is known to be a cell adhesion molecule. Actual localization of these proteins remains to be elucidated in future studies by, for instance, expressing the green fluorescence protein (GFP)-fusion protein together with using the transformation technique presented in this study.

A unique gene family, L-lactate permease (PF02652), is responsible for uptake of lactate in the cells. Three predicted genes (GENE_00005863-RA, GENE_00005866-RA, and GENE_00005870-RA) encoding lactate permease exist in proximity on contig 5 along with GENE_00005868-RA which were not assigned into any functional family (Supplementary Fig. S7). To our knowledge, lactate utilization in green algae has not be intensively studied, whereas heterotrophic/mixotrophic cultivation of diatoms, which are microalgae in other taxonomic groups, using lactate has long been studied^30^. Interestingly, homology search with BLASTp revealed that the proteins encoded by the aforementioned 4 genes showed sequence similarity with that of lactate permease of diatoms including *Phaeodactylum tricornutum*, while the proteins of *Chlorococcum* sp. FFG039 were like the split forms of that of *Phaeodactylum tricornutum* (Supplementary Fig. S7). It remains elusive whether these proteins are actually expressed in the split forms as predicted. If these proteins function as active lactate permeases, *Chlorococcum* sp. FFG039 might be capable of utilization of lactate because it has the genes encoding lactate/malate dehydrogenase; which can convert lactate to pyruvate, within the genome. It has been reported that *Bacillus subtilis* expressed lactate permease and utilized lactate as an alternative carbon source, only when the biofilms were formed^31^. Although little is known about the relationship between lactate metabolism and biofilm formation in microalgae, we assume that lactate permeases found in the genome of *Chlorococcum* sp. FFG039 might be related to the phenotype of biofilm formation. Further study is needed to conclude the availability of lactate for this microalga.

In conclusion, a genetic transformation technique based on electroporation was established and the genome analysis was performed for the analysis of a water surface-floating microalgae, *Chlorococcum* sp. FFG039. Insertion of an antibiotic-resistant gene, *nptII* into the genomic DNA was confirmed by PCR and southern hybridization. The expression of *nptII* was achieved with heterogeneous promoters, namely the 35 S subunit-promoter derived from CaMV or RNA polymerase α subunit-promoter derived from a diatom. It was confirmed that the obtained antibiotic-resistance phenotype was retained for at least 5 months. Genome sequencing using next generation sequencing technique highlighted the genomic landscape of the nuclear genome with a size of approximately 72 Mb, containing 20,615 predicted genes. Comparison of the genomes of *Chlorococcum* sp. FFG039 and other 4 species of non-floating green microalgae revealed the unique gene families of *Chlorococcum* sp. FFG039, some of which may be involved in biofilm formation and water surface-floating abilities. These techniques and information will be useful for future studies to elucidate molecular mechanism of the water surface-floating phenotype of *Chlorococcum* sp. FFG039.

## Materials and Methods

### Strain and culture conditions

A green microalga, *Chlorococcum* sp. FFG039 was maintained in modified-CSi medium (750 mg Ca(NO_3_)_2_·4H_2_O, 500 mg KNO_3_, 142 mg K_2_HPO_4_, 200 mg MgSO_4_·7H_2_O, 15 mg Na_2_EDTA, 111 mg KH_2_PO_4_, 0.5 µg vitamin B_12_, 0.5 µg biotin, 50 µg thiamine HCl, 2.94 mg FeCl_3_·6H_2_O, 540 µg MgCl_2_·4H_2_O, 330 µg ZnSO_4_·7H_2_O, 60 µg CoCl_2_·6H_2_O, 37.5 µg Na_2_MoO_4_·2H_2_O per litter of distilled water, pH 6.0) under continuous illumination using cool white fluorescent lights (40 µmol photons/m^2^/s, 25 °C) with shaking (125 rpm). Modified-CSi medium containing 1% agarose (Funakoshi, Tokyo, Japan) was used for colony formation. To obtain the floating biomass, *Chlorococcum* sp. FFG039 was cultured in 40 ml-volume plastic cases (size: 50 (H) × 63 (W) × 25 (D) mm^3^, AS ONE, Osaka, Japan) in a desiccator (AS ONE, Osaka, Japan), without shaking, with initial cell density of 1 × 10^5^ cells/ml. The cells in the plastic cases were grown at 25 °C under continuous cool white fluorescent lights at 200 μmol photons /m^2^/s.

### Antibiotic sensitivity of *Chlorococcum* sp. FFG039

For establishment of transformation technique, *neomycin phosphotransferase II* (*nptII*) was transferred into *Chlorococcum* sp. FFG039. Transformants were screened by culturing them with antibiotic G418 (Roche Applied Science Co. LLC., Penzberg, Germany), at appropriate concentration determined as described below. *Chlorococcum* sp. FFG039 was cultured in modified CSi liquid medium or that containing 1% agarose with various concentrations of G418. In the liquid medium, the cells were cultured for 2 weeks with 0, 50, 100, 250, 500, or 1000 µg/ml of G418 under the shaking condition (125 rpm) where the cells do not form biofilm and remain suspended. The cell growth was evaluated by measurement of optical density at 750 nm (OD_750 nm_). In an additional experiment, 1.0 × 10^3^ cells were cultured on the agar medium, with 0, 2.5, 5.0, 10, 20 or 30 µg/ml of G418 for 2 weeks (70 µmol/m^2^/s, 25 °C). Subsequently, colonies formed on the agar medium were counted. Colony formation efficiency was calculated using the following Eq. (1).

1 $${\rm{Colony}}\,{\rm{formation}}\,{\rm{efficiency}}\,( \% )=\frac{{\rm{Number}}\,{\rm{of}}\,{\rm{G}}418\,{\rm{resistant}}\,{\rm{colonies}}}{{\rm{Number}}\,{\rm{of}}\,{\rm{plated}}\,\mathrm{cells}\,(1.0\times {10}^{3}\,{\rm{cells}})}\times 100\,$$

### Electroporation

*NptII*-expression vectors, pSP-NPT/CaMV and pSP-NPT/rpoA were constructed in our previous study^15^. The 35 S subunit-promoter derived from CaMV or RNA polymerase α subunit-promoter derived from a diatom *Fistulifera solaris*, was used for expression of *nptII*. The cells were cultured in Erlenmeyer flasks containing 40 ml of the modified CSi medium. Cultivation was performed with shaking the flasks (125 rpm) to avoid biofilm formation, until the culture reached the exponential phase. The cultured cells were collected by centrifugation (8,500 *g*, 15 min, 15 °C), and washed twice with ultrapure water. Thereafter the cells were re-suspended in ultrapure water at 1.0 × 10^8^ cells/ml, and 250 µl of the cell suspension was transferred to each tube. pSP-NPT/CaMV or pSP-NPT/rpoA was added to the cell suspension, and incubated on ice for 15 min. Subsequently, electroporation was performed using Gene Pulser Xcell (Bio-Rad, Hercules, California, U.S.A.). In order to optimize the electroporation conditions, we changed the applied voltage (1.0, 2.0, 4.0, 6.0, 8.0, and 10.0 kV/cm), gap of electrodes in the cuvette (0.2 and 0.4 cm), and amount of plasmids introduced to the cell samples (1.0, 2.5, 5.0, and 10 µg). After electroporation was performed, the resultant cells were suspended in 2.5 ml of the modified CSi medium, and incubated overnight. Then, the cells were spread on the agar medium with 20 µg/ml G418 (100 µl of suspension for each agar medium plate), and cultured (70 µmol/m^2^/s, 25 °C) for at least 3 weeks, followed by colony counting.

### Southern hybridization

The G418-resistant colonies were picked from the agar medium with 20 µg/ml G418, and cultured in 96-well plates containing modified CSi medium without G418 for 2 weeks, followed by scaling-up to 10 ml. Genomic DNA was extracted from the cultured cells using NucleoSpin Tissue XS (Takara Bio Inc., Shiga, Japan) or NucleoBond Buffer Set III and Nucleo BondAXG100 (Takara Bio Inc., Shiga, Japan). Insertion of *nptII* was first confirmed by PCR with the primer set 5′-ATG ATT GAA CAA GAT GGA TTG C-3′ and 5′-TCA GAA CTC GTC AAG AAG G-3′. Subsequently, 10 µg of genomic DNA was digested using restriction enzymes *Bgl*II and *Sal*I. The digested DNA was subjected to electrophoresis using a 0.8% agarose gel, and transferred to Amersham Hybond-N+ membrane (GE Healthcare UK Ltd., Buckinghamshire, UK) using 20× saline sodium citrate (SSC) buffer, followed by depurination, denaturation and neutralization. Biotin-labelled probe for hybridization was synthesized using the total length of *nptII* fragment (795 bp) amplified by PCR and North2South Biotin Random Prime Labeling kit (Thermo Fisher Scientific Inc., Waltham, MA). The probe hybridizing with the genomic DNA on the membrane was detected using North2South Chemiluminescent Hybridization and Detection kit (Thermo Fisher Scientific Inc.), and C-Digit Blot Scanner (LI-COR Biosciences Inc., Lincoln, NE, USA).

### Genome extraction and sequencing

Prior to genome analysis of *Chlorococcum* sp. FFG039, we prepared the medium agar plates for colony formation. A single colony was collected from the plate, and subsequently, the cells in the colony was transferred to the modified CSi liquid medium containing a cocktail of antibiotics (50 μg/ml ampicillin, 50 μg/ml kanamycin, 50 μg/ml streptomycin, 50 μg/ml gentamycin, and 2 μg/ml chloramphenicol) to avoid bacterial contamination. We repeated this process thrice, thereafter we amplified 18 S rRNA gene by PCR (5′-GGT GAT CCT GCC AGT CAT ATG CTT G-3′ and 5′-GAT CCT TCC GCA GGT TCA CCT ACG GAA ACC-3′), and confirmed that the sequence of the amplified fragment showed high sequence identity with that of *Chlorococcum sp*. RK261. The as-prepared seed culture was used for cultivation in flat flasks containing the modified CSi medium along with cocktail of antibiotics and air bubbling with 2% CO_2_ at 0.8 L/L/min, at 25 °C, under 160 μmol photons/m^2^/s for 10 days. The cells were collected by centrifugation (6,300 *g*, 10 min), and frozen with liquid nitrogen. The frozen cells (approximately 5 × 10^9^ cells) were homogenized using a mortar and pestle. Subsequently, genomic DNA was extracted using NucleoBond Buffer Set III and Nucleo BondAXG100 (Takara Bio Inc., Shiga, Japan) as per the manufacture’s instruction. The SMRT Sequencing with PacBio RSII system using P6/C4 chemistry was performed in Macrogen, Inc. (Seoul, Rep. of Korea).

### Bioinformatics

*De novo* assembly of the sequences were performed with Canu (ver. 1.4). Genome annotation was carried out with Maker pipeline (ver. 2.31.8) and BLAST+ (ver. 2.4.0, E-value: 1e-3, database: NT and NR). Manipulation of FASTA files and calculation of GC content were performed with SeqKit^32^. Genomic landscapes for nuclear and organelle genomes were visualized with ClicO FS^33^ and GeSeq^34^, respectively. Dot-plot analysis to compare two genomes were performed using D-GENIES with the option of minimap2^35^. List of gene families were generated with InterProScan 5.23–62.0 and amino acid sequences encoded by the predicted genes. Subcelullar localization of the proteins of interest were predicted with DeepLoc^27^.

## Supplementary information

Supplementary Information
