# Supplementary material for: Genome analysis and genetic transformation of a water surface-floating microalga Chlorococcum sp. FFG039
Source: Sci Rep. 2019 Aug 1;9:11200. doi: 10.1038/s41598-019-47612-8 (PMC6671960; doi:10.1038/s41598-019-47612-8)
Supplement: Supplementary file 1 — Supplementary Information [file 41598_2019_47612_MOESM1_ESM.pdf]

Supplementary Information for:

Genome analysis and genetic transformation of a water surface-floating microalga *Chlorococcum* sp. FFG039

†Yoshiaki Maeda[a], †Daisuke Nojima[a], Miki Sakurai[a], Tatsuhiro Nomaguchi[b], Momoko Ichikawa[a], Yuki Ishizuka[a] and [*]Tsuyoshi Tanaka[a]

[a]Division of Biotechnology and Life Science, Institute of Engineering, Tokyo University of Agriculture and Technology, 2-24-16 Naka-cho, Koganei, Tokyo, Japan, 184-8588

[b]Department of Advanced Science and Engineering, Graduate School of Advanced Science and Engineering, Waseda University, 3-4-1 Okubo, Shinjuku-ku, Tokyo, Japan, 169-8555

† Equal contribution

*Corresponding author

E-mail address: tsuyo@cc.tuat.ac.jp

Tel: +81-42-388-7401

Fax: +81-42-385-7713

Supplementary Table S1 Colony formation of *Chlorococcum* sp. FFG039 on the modified CSi agar medium containing various concentrations of G418

Concentration of G418 (μg/ml)	Number of colonies	Colony formation efficiency (%)[a]
0	288 ± 92	28.8
2.5	219 ± 36	21.9
5	282 ± 136	28.2
10	151 ± 63	15.1
20	0	0
30	0	0

[a] Colony formation efficiency (%) = number of the G418-resistant colonies/number of the plated cells (10^3 cells)

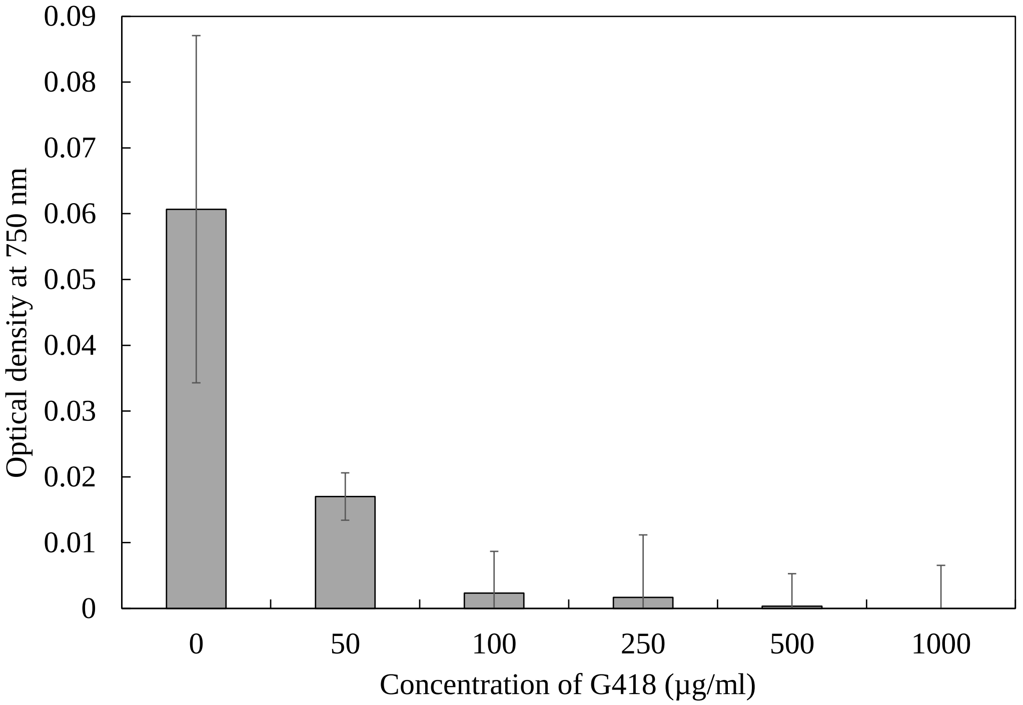

Supplementary Fig. S1 Growth of *Chlorococcum* sp. FFG039 in the modified CSi liquid medium containing various concentrations of G418. Optical density at 750 nm was measured after 2 weeks.

Supplementary Table S2 Formation of G418-resistant colonies of *Chlorococcum* sp. FFG039 after electroporation using 2.5×10^7 cells, pSP-NPT/CaMV or pSP-NPT/rpoA (5.0 μg) and 0.2 cm cuvettes under various applied voltage. The experiment was repeated twice.

Applied voltage (kV/cm)	Average number of G418 resistant colonies per 10^6 cells		
	Prompter	35S	rpoa
1.0		3 (0, 6)	10 (0, 19)
2.0		18 (0,36)	2 (0, 3)
4.0		3 (0, 5)	6 (1, 11)
6.0		1 (0, 1)	1 (0, 2)
8.0		1 (1, 0)	1 (1, 0)
10.0		1 (0, 1)	3 (4, 1)

Supplementary Table S3 Formation of G418-resistant colonies of *Chlorococcum* sp. FFG039 after electroporation using 2.5×10^7 cells, various amounts of pSP-NPT/CaMV or pSP-NPT/rpoA and 0.2 cm cuvettes under the applied voltage of 2.0 kV/cm. The experiment was repeated five times.

Amount of plasmid (μg)	Average number of G418 resistant colonies per 10^6 cells		
	Promoter	35S	rpoa
1.0		49 (6, 82, 150, 0, 5)	18 (10, 17, 60, 1, 0)
2.5		51 (4, 101, 152, 0, 0)	56 (12, 37, 217, 14, 0)
5.0		43 (0, 68, 146, 0, 2)	11 (6, 25, 24, 0, 0)
10.0		15 (14, 26, 37, 0, 0)	34 (9, 61, 98, 0, 0)

Supplementary Table S4 Formation of G418-resistant colonies of *Chlorococcum* sp. FFG039 after electroporation using 2.5×10^7 cells, pSP-NPT/CaMV or pSP-NPT/rpoA (2.5 μg) and 0.2 or 0.4 cm cuvettes under the applied voltage of 2.0 kV/cm. The experiment was repeated thrice.

Gap size (cm)	Average number of G418 resistant colonies per 10^6 cells		
	Promoter	35S	rpoa
0.2		18 (45, 9, 0)	17 (46, 5, 0)
0.4		41 (46, 11, 65)	34 (26, 12, 66)

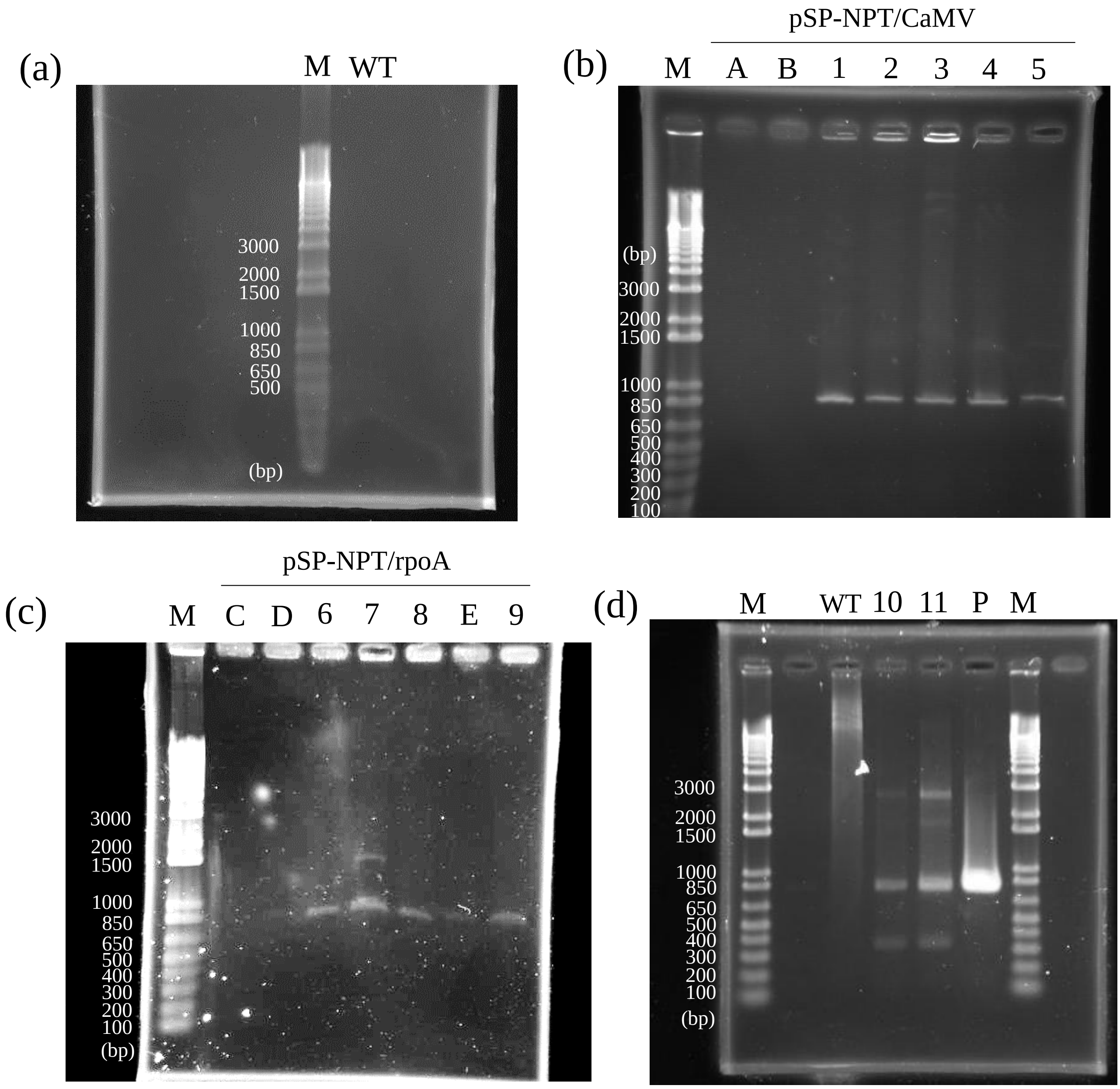

Supplementary Fig. S2 Electrophoresis of PCR products targeting *nptII* gene (795 bp). *Chlorococcum* sp. FFG039 was transformed by electroporation with pSP-NPT/CaMV or pSP-NPT/rpoA. M stands for molecular marker. WT stands for wild type as negative control (a and d). The PCR products were amplified from the extracted genomic DNA of WT and G418-resistant transformant clones cultured for 2 weeks (a-c) and 5 months (d). Lane 1-5 and 10: representative clones with pSP-NPT/CaMV (b and d), Lane 6-9 and 11: representative clones with pSP-NPT/rpoA (c and d). Lane A-E: the clones from which amplification was not clearly confirmed. Lane P: positive control using the purified plasmid as a template.

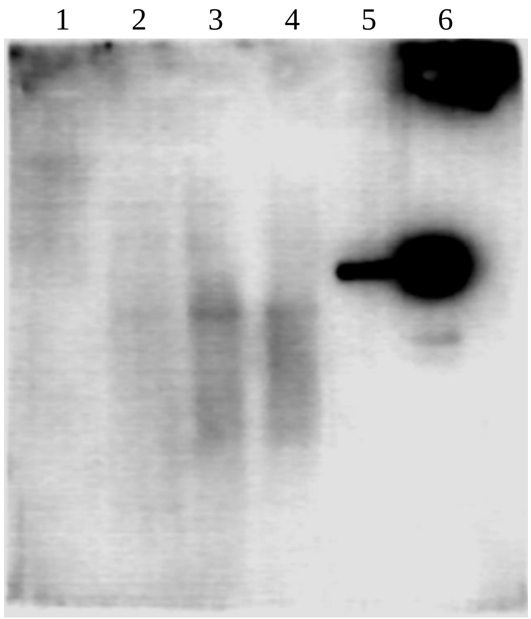

Supplementary Fig. S3 Southern hybridization targeting the whole sequence of *nptII* (795 bp). *Chlorococcum* sp. FFG039 was transformed by electroporation, and gene retention and stability were confirmed.

Lane 1: Wild type, Lane 2: Transformants with pSP-NPT/CaMV, Lane 3 and 4: pSP-NPT/H4 [1], Lane 5: Purified pSP-NPT/CaMV (1 ng, positive control, ~4.1 kbp), Lane 6: Purified pSP-NPT/CaMV (10 ng, positive control)

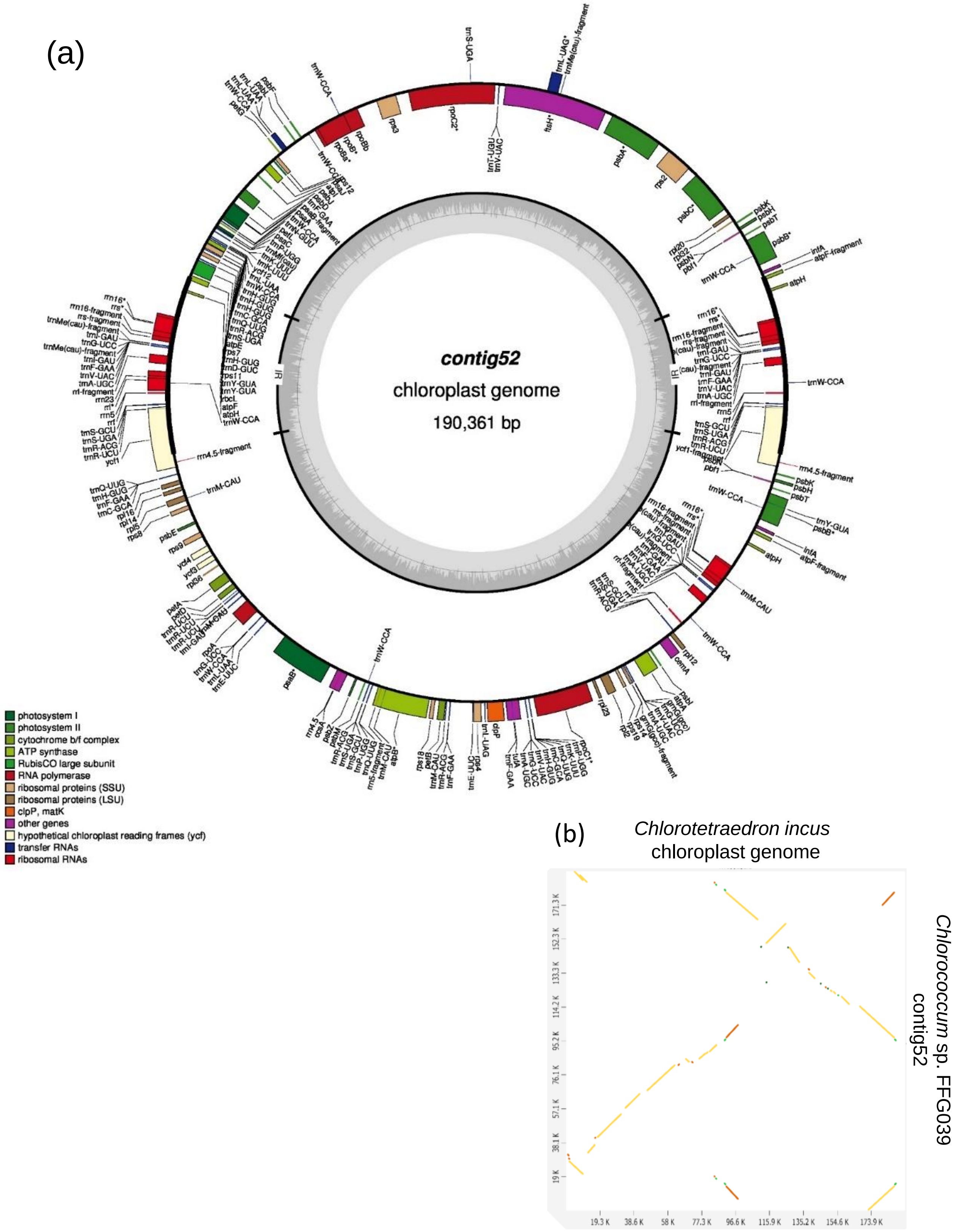

Supplementary Fig. S4 Map of contig 52 corresponding to the chloroplast genome of *Chlorococcum* sp. FFG039. (a) The map was drawn using GeSeq with the reference sequences of chloroplast genomes of *Chlorotetraedron incus* and *Neochloris aquatic*. (b) Dot-plot analysis to compare the chloroplast genomes of *Chlorococcum* sp. FFG039 and *Chlorotetraedron incus*

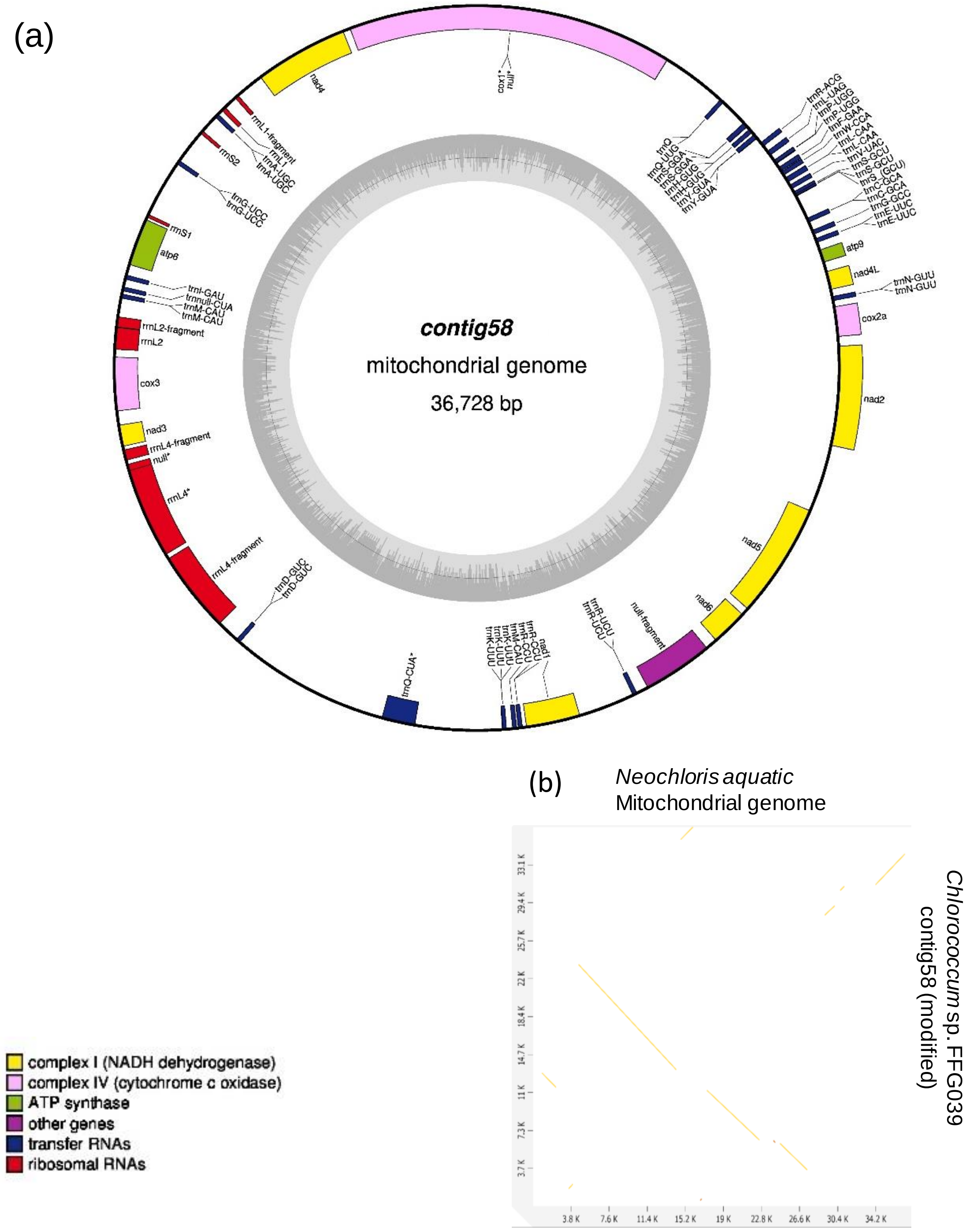

Supplementary Fig. S5 Map of contig 58, in which duplicated parts were trimmed, corresponding to the mitochondrial genome of *Chlorococcum* sp. FFG039. (a) The map was drawn using GeSeq with the reference sequences of mitochondrial genomes of *Chlorotetraedron incus* and *Neochloris aquatic*. (b) Dot-plot analysis to compare the mitochondrial genomes of *Chlorococcum* sp. FFG039 and *Neochloris aquatic*

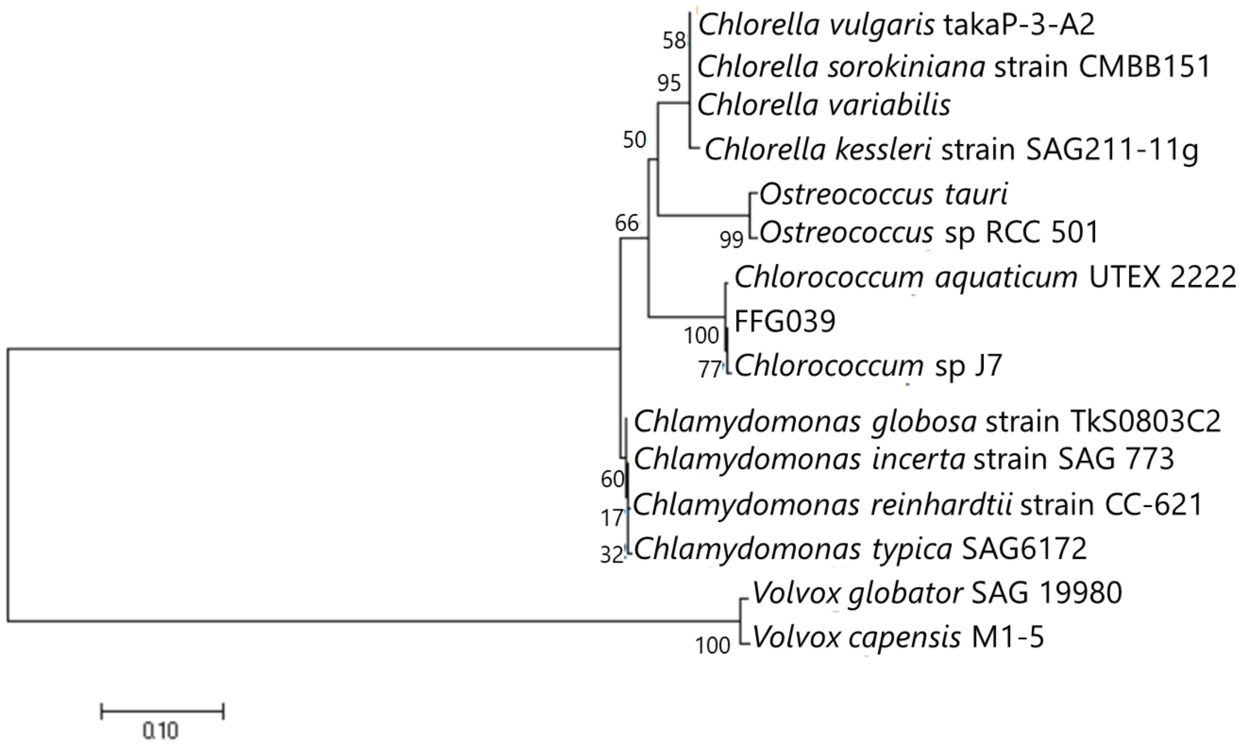

Supplementary Fig. S6 Phylogenetic relationship of the green algae including *Chlorococcum* sp. FFG039, *Chlamydomonas reinhardtii*, *Osteococcus tauri*, *Chlorella variabilis*, *Volvox cateri* and the closely-related species. Phylogenetic tree was constructed with 18S rRNA gene sequences using the neighbour-joining method (bootstrap = 1000) on MEGA7.

Supplementary Table S5 Unique gene families of *Chlorococcum* sp. FFG039 as compared to those of *Chlamydomonas reinhardtii*, *Osteococcus tauri*, *Chlorella variabilis*, and *Volvox cateri*. The gene families mentioned in the main text are highlighted in yellow.

Pfam ID	description	number of genes
PF11380	Stealth protein CR2, conserved region 2	4
PF14310	Fibronectin type III-like domain	1
PF07463	NUMOD4 motif	1
PF13392	HNH endonuclease	6
PF10138	vWA found in TerF C terminus	2
PF05142	Domain of unknown function (DUF702)	1
PF12657	Transcription factor IIIC subunit delta	1
PF07679	Immunoglobulin I-set domain	1
PF14783	Ciliary BBSome complex subunit 2, middle region	1
PF00084	Sushi repeat (SCR repeat)	5
PF08706	D5 N terminal like	6
PF16919	Protein kinase G rubredoxin domain	1
PF08647	BRE1 E3 ubiquitin ligase	1
PF12872	OST-HTH/LOTUS domain	1
PF06724	Domain of Unknown Function	1
PF08740	BCS1 N terminal	2
PF06405	Red chlorophyll catabolite reductase	1
PF04991	LicD family	2
PF13582	Metallo-peptidase family M12B	1
PF04547	Calcium-activated chloride channel	3
PF08707	Primase C terminal 2 (PriCT-2)	6
PF11160	Protein of unknown function (DUF2945)	1
PF09924	Uncharacterised conserved protein (DUF2156)	4
PF03131	bZIP Maf transcription factor	1
PF02040	Arsenical pump membrane protein	2
PF08883	Dopa 4,5-dioxygenase family	3
PF16884	N-terminal domain of oxidoreductase	1
PF08144	CPL (NUC119) domain	1
PF13430	Domain of unknown function (DUF4112)	2
PF14114	Domain of unknown function (DUF4286)	1
PF16858	Condensin II complex subunit CAP-H2 or CNDH2, C-term	1
PF02838	Glycosyl hydrolase family 20, domain 2	1
PF13452	N-terminal half of MaoC dehydratase	1
PF10160	Predicted membrane protein	1
PF05225	helix-turn-helix, Psq domain	1
PF04166	Pyridoxal phosphate biosynthetic protein PdxA	1
PF12831	FAD dependent oxidoreductase	2
PF02652	L-lactate permease	3
PF02399	Origin of replication binding protein	2
PF14587	O-Glycosyl hydrolase family 30	1
PF07168	Ureide permease	1
PF13528	Glycosyl transferase family 1	1
PF02979	Nitrile hydratase, alpha chain	2
PF02211	Nitrile hydratase beta subunit	2
PF14771	Domain of unknown function (DUF4476)	3
PF06772	Bacterial low temperature requirement A protein (LtrA)	1
PF13892	DNA-binding domain	1
PF05033	Pre-SET motif	1
PF16862	Glycosyl hydrolase family 79 C-terminal beta domain	1
PF10282	Lactonase, 7-bladed beta-propeller	1
PF13620	Carboxypeptidase regulatory-like domain	4
PF00779	BTK motif	1
PF15236	Coiled-coil domain-containing protein 66	1
PF14817	HAUS augmin-like complex subunit 5	1
PF05994	Cytoplasmic Fragile-X interacting family	1
PF01969	Protein of unknown function DUF111	1

continued

Pfam ID	description	number of genes
PF02487	CLN3 protein	2
PF14397	Sugar-transfer associated ATP-grasp	1
PF04689	DNA binding protein S1FA	1
PF14534	Domain of unknown function	1
PF12972	Alpha-N-acetylglucosaminidase (NAGLU) C-terminal domain	1
PF05089	Alpha-N-acetylglucosaminidase (NAGLU) tim-barrel domain	1
PF12971	Alpha-N-acetylglucosaminidase	1
PF11705	DNA-directed RNA polymerase III subunit Rpc31	1
PF12357	Phospholipase D C terminal	1
PF00614	Phospholipase D Active site motif	1
PF10186	Vacuolar sorting 38 and autophagy-related subunit 14	1
PF09248	Domain of unknown function (DUF1965)	1
PF10198	Vacuolar sorting 38 and autophagy-related subunit 14	1
PF12036	Protein of unknown function	1
PF17123	RING-like zinc finger	3
PF14739	Domain of unknown function (DUF4472)	1
PF03492	SAM dependent carboxyl	2
PF01483	Proprotein convertase P-domain	1
PF02434	Fringe-like	2
PF13475	Domain of unknown function	3
PF10712	NAD-specific glutamate dehydrogenase	2
PF14324	PINIT domain	1
PF06495	Fruit fly transformer protein	1
PF00008	EGF-like domain	2
PF04413	3-Deoxy-D-manno-octulosonic-acid transferase (kdotransferase)	1
PF00066	LNR domain	1
PF14932	HAUS augmin-like complex subunit 3	1
PF06209	Cofactor of BRCA1 (COBRA1)	1
PF02224	Cytidylate kinase	1
PF14774	FAM177 family	1
PF10544	T5orf172 domain	1
PF13577	SnoaL-like domain	1
PF09767	Predicted membrane protein	1
PF04571	lipin, N-terminal conserved region	1
PF04750	FAR-17a/AIG1-like protein	1
PF00068	Phospholipase A2	1
PF04239	Protein of unknown function (DUF421)	1
PF05426	Alginate lyase	1
PF00998	Viral RNA dependent RNA polymerase	1
PF02311	AraC-like ligand binding domain	1
PF14531	Kinase-like	1
PF03406	Phage tail fibre repeat	1
PF13358	DDE superfamily endonuclease	1
PF01419	Jacalin-like lectin domain	1
PF03352	Methyladenine glycosylase	1
PF00942	Cellulose binding domain	1
PF04199	Putative cyclase	1
PF11336	Protein of unknown function	1
PF06441	Epoxide hydrolase N terminus	1
PF11995	Domain of unknown function (DUF3490)	2
PF10347	RNA pol II promoter Fmp27 protein	1
PF06397	Desulfoferrodoxin, N-terminal domain	1
PF14464	Prokaryotic homologs of the JAB	1
PF14777	Cilia BBSome complex subunit 10	1
PF05837	Centromere protein H (CENP-H)	1
PF09262	Peroxisome biogenesis factor 1, N-terminal	1
PF09347	Domain of unknown function (DUF1989)	1

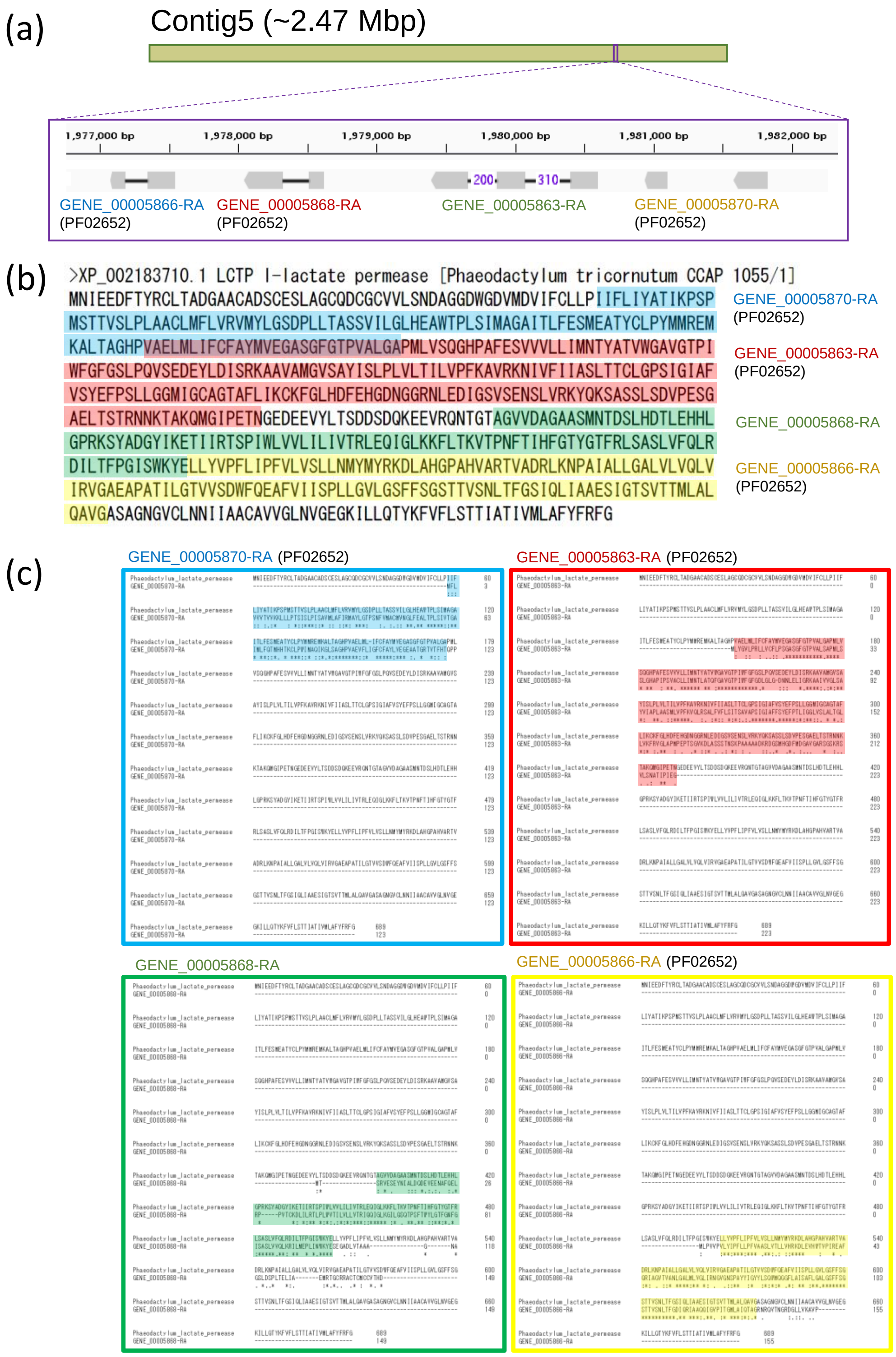

Supplementary Fig. S7 L-lactate permeases (GENE_00005863-RA, GENE_00005866-RA, and GENE_00005870-RA) of *Chlorococcum* sp. FFG039. (a) The genes encoding lactate permease exist in proximity on contig 5 along with GENE_00005868-RA, which were not assigned to any functional family. (b) Lactate permeases of a diatom *Phaeodactylum tricornutum* showing sequence similarity to those in *Chlorococcum* sp. FFG039. (c) Alignment results of L-lactate permeases of *P. tricornutum* and *Chlorococcum* sp. FFG039 with Clustal Omega.

Reference

1 Muto, M. *et al.* Establishment of a genetic transformation system for the marine pennate diatom *Fistulifera* sp. strain JPCC DA0580--a high triglyceride producer. *Mar. Biotechnol.* **15**, 48-55 (2013).
